# MicroRNAs modulated by DPP-4 inhibitor and bedtime NPH insulin therapy in individuals with type 2 diabetes

**DOI:** 10.3389/fendo.2025.1706951

**Published:** 2025-11-07

**Authors:** Aritania Sousa Santos, Silvia Yumi Bando, Maria Lucia Cardillo Correa Giannella, Maria Elizabeth Rossi da Silva

**Affiliations:** 1 Laboratório de Carboidratos e Radioimunoensaio (LIM18), Hospital das Clínicas (HCFMUSP), Faculdade de Medicina, Universidade de Sao Paulo, São Paulo, Brazil; 2 Department of Pediatrics, Faculdade de Medicina da Universidade de São Paulo, São Paulo, Brazil

**Keywords:** basic clinical research, miRNAs, DPP-4 inhibitor, insulin, type 2 diabetes

## Abstract

**Background:**

MicroRNAs (miRNAs) and their target genes can elucidate mechanisms of drug action and serve as potential therapeutic biomarkers.

**Methods:**

To evaluate the effects of bedtime NPH insulin and sitagliptin on serum miRNA expression in individuals with type 2 diabetes (T2D), thirty-two patients with T2D inadequately controlled with metformin and glyburide were randomly assigned to an additional 6-month treatment with either bedtime NPH insulin or sitagliptin. Before and after treatments, fasting as postprandial (60, 120, and 180 minutes) concentrations of glucose, C-peptide, glucagon-like peptide 1 (GLP1), and triglycerides were measured. Fasting HbA1c was also assessed. Expression levels of selected miRNAs were analyzed using quantitative polymerase chain reaction.

**Results:**

The sitagliptin and bedtime NPH insulin groups were comparable in age, body mass index, diabetes duration, and baseline metabolic variables. Both treatments led to a similar reduction in HbA1c. Only sitagliptin increased postprandial GLP1 concentrations. Sitagliptin treatment upregulated six miRNAs: miR-24-3p, miR-27a-3p, miR92a-3p, let-7d-5p, miR-30c-5p, and miR-660-5p. NPH insulin upregulated four miRNAs: miR-92a-3p, miR-193b-3p, miR-320a-3p and miR-30c-5p. Both treatments increased miR-92a-3p and miR-30c-5p, particularly at fasting and 60 minutes post-meal. KEGG pathways analysis revealed enrichment in signaling pathways related to insulin action, growth/development, cellular senescence, lipid/atherosclerosis, Th17 cell differentiation, insulin resistance, autophagy, and apoptosis. Sitagliptin and bedtime NPH insulin induced metabolic improvement and distinct modulation of circulating miRNAs, with sitagliptin influencing a broader spectrum of miRNA expression.

**Conclusion:**

The upregulated miRNAs are involved in pathways related to insulin signaling, inflammation, and cellular homeostasis and support the hypothesis that sitagliptin exerts pleiotropic effects beyond glycemic control.

## 1 Introduction

Type 2 diabetes (T2D) is a heterogeneous metabolic disorder characterized by chronic hyperglycemia and associated with severe microvascular, macrovascular and neurological complications that significantly reduce both quality of life and life expectancy. The disease results from a combination of genetic and environmental factors, particularly sedentary behavior and high-calorie diets rich in fat and processed foods, which contribute to insulin resistance and progressive pancreatic beta-cell dysfunction. Management of T2D involves lifestyle interventions – including calorie restriction and physical activity, and pharmacotherapy (1).

Sulfonylureas, which stimulate insulin secretion, and metformin, which reduces hepatic gluconeogenesis and improves insulin sensitivity, are widely used to treat patient with T2D due to their efficacy and low cost (1).

However, when these therapies fail to achieve glycemic targets, selecting a third-line medication remains a challenge, especially with regard to its effectiveness in improving metabolic control and preventing complications.

Both bedtime NPH insulin and incretin-based therapy with dipeptidyl peptidase 4 (DPP4) inhibitors are recommended by the American Diabetes Association for use in selected populations, particularly in older adults and individuals in low-resource settings. DPP4 inhibitors, also known as gliptins, are oral agents that prevent the degradation of glucagon-like peptide 1 (GLP1). By increasing GLP1 bioavailability, they exert several glucose-lowering effects: stimulation of insulin secretion in a glucose-dependent manner, reduction of postprandial glucagon, inhibition of hepatic glucose production, and enhancement of peripheral glucose uptake, mainly in the post-prandial period (1, 2). In addition to glycemic benefits, DPP4 inhibitors have been shown to improve cardiovascular risk factors, including left ventricular diastolic dysfunction, independently of glycemic control, as previously demonstrated by our group (3). Conversely, bedtime NPH insulin primarily targets fasting hyperglycemia by decreasing hepatic glucose output overnight and promoting cellular glucose uptake (1).

Identifying biomarkers that predict therapeutic response could support individualized treatment decisions. In this context, microRNAs (miRNAs) have emerged as promising candidates. These small, non-coding RNAs function as post-transcriptional negative regulators of gene expression and are involved in a wide range of biological processes, including development, cell proliferation, differentiation, apoptosis, and metabolic regulation (4, 5). They may also provide insights into drug mechanisms of action by revealing the pathways associated with their target genes.

Recent studies have reported associations between antidiabetic therapies and changes in miRNA expression profiles following treatment with metformin (6), tiazolidinediones (7), DPP4 inhibitors (8), intensive insulin therapy (9), and GLP1 receptor agonists (10) Notably, each drug class appears to modulate a distinct and limited set of miRNAs.

Circulating miRNAs have been proposed as biomarkers of T2D risk and therapeutic efficacy (10). However, it remains unclear whether the pharmacological effects of these drugs are mediated by miRNAs, or whether miRNA changes simply reflect improvements in metabolic variables.

In a previous study, we compared the efficacy of the DPP4 inhibitor sitagliptin *versus* bedtime NPH insulin as add-on therapy in T2D individuals inadequately controlled with metformin and glyburide (11). Building upon those findings, the present study aimed to investigate whether treatment with sitagliptin or bedtime NPH insulin was associated with changes in circulating miRNA expression profiles, related to T2D and/or insulin resistance: miR-24-3p, miR30c-5p (4), miR-193b-3p (12), miR-335-5p (13), miR-199a-3p, miR-532-5p, let-7d-5p (14), miR-27a-3p (15), miR-660-5p (16), miR-92a-3p and miR-320-3p (6). Their participation in pathways related to metabolism and diabetes are presented in **Supplementary Table 1**. We further examined whether these changes were linked to improvements in metabolic control, insulin, glucagon, and GLP1 concentrations. We also explored the potential biological relevance of the differentially expressed miRNAs by analyzing their predicted target genes involved in pancreatic hormone secretion and insulin resistance pathways.

## 2 Materials and methods

The protocol adhered to the Declaration of Helsinki and was approved by the Ethics Committee of Hospital das Clínicas, Faculdade de Medicina, Universidade de Sao Paulo (HCFMUSP) under approval numbers 415/01 and 0215/08. Written informed consent was obtained from all participants.

In a previous prospective study, 35 outpatients with T2D inadequately controlled with metformin plus glyburide were randomized in parallel to receive an additional 6-month treatment with either bedtime NPH insulin (NPH group) or 100 mg of sitagliptin once daily (SITA group). Exclusion criteria included: heart or respiratory failure, uncontrolled hypertension, hepatic, renal, endocrine and gastrointestinal disorders, malignancy, alcohol abuse, and prior use of insulin or incretin-based therapy.

Participants were followed weekly for drug and dietary adjustments during the first month and then monthly for six-month. Baseline clinical data, anthropometric and laboratory assessments were collected at enrollment (pre-treatment) and repeated at the end of the 6-month intervention.

### 2.1 Laboratory procedures

The participants were instructed to follow a consistent diet and abstain from alcohol, caffeine and vigorous physical activity 24 hours before testing. After a 12-hour overnight fast, a standardized 500-kcal mixed meal (60% carbohydrate, 20% fat, 20% protein) was administered. Blood samples were collected at 0, 60, 120 and 180 minutes for measurement of glucose, insulin, glucagon, proinsulin, C-peptide, active GLP1 (aGLP1), free fatty acids, and triglycerides. Fasting cholesterol and HbA1c values were also assessed. The study utilized laboratory and demographic data previously reported in a evaluation of 6- and 12- month SITA or NPH insulin treatments (11), which are presented in **Supplementary Table 2** and Figures.

### 2.2 Sample collection and quality control

Blood samples were collected in tubes containing separation gel (Becton Dickinson) to obtain serum, which was then stored in a freezer at -80°C until total RNA extraction.

### 2.3 Assessment of hemolysis

Initially, the samples were evaluated for hemolysis using spectrophotometry (NanoDrop 2000 spectrophotometer, Thermo Scientific, Waltham, Massachusetts, USA), with absorbance measured between 350 and 650 nm. The degree of hemolysis was determined based on the optical density (OD) at 414 nm—the absorbance peak of free hemoglobin. Additional peaks at 541 and 576 nm were also monitored, as they are indicative of a higher degree of hemolysis. Samples were classified as “hemolyzed” if the OD at 414 nm exceeded 0.2 (17). Hemolyzed samples were excluded from the study.

### 2.4 Extraction of total serum RNA enriched in miRNAs

Total RNA enriched in miRNAs was extracted from 200 μL of serum using the miRNeasy Serum/Plasma kit (Qiagen, Germany) following the manufacturer’s instructions. A synthetic spike-in control (cel-miR-39, *C. elegans*, 2.0 μL of the 2.5x10–^5^ pmoL/μL) was added to each sample to allow normalization of technical variation and was used as the external reference miRNA. RNA was eluted with 14 μL of RNase-free water and quantified by absorbance at 260/280 nm using a NanoDrop ND-2000 apparatus (Thermo Scientific, USA). Samples were stored at −80°C until analysis.

### 2.5 MiRNA expression profiling

Ten candidates’ miRNAs were selected for expression analysis: hsa-24-3p, hsa-27a-3p, hsa-92a-3p, hsa-let-7d-5p, hsa-199a-3p, hsa-335-5p, has-532-5p, hsa-miR-193b-3p, hsa-miR-660-5p, hsa-miR-30c-5p, described at **Supplementary Table 3**. The external miRNA cel-miR-39-3p was used as a reference gene to normalize miRNA expression data (18).

#### 2.5.1 Reverse transcription reaction

We used the TaqMan™ Advanced miRNA cDNA Synthesis Kit (A28007), comprising 4 steps (manufacturer’s protocol): Step 1: Poly (A) tailing reaction – 10X Poly(A) Buffer (0.5µL); ATP (0.5µL); Poly (A) Enzyme 0.3 µL, RNAse-free water (1.7 µL) and 2 µL of the RNA sample, according to the following Veriti thermocycler cycling (Applied Biosystems- Thermofisher): Polyadenylation 37°C for 45 minutos; Stop reaction 65°C for 10 minutes and finished at 4°C. Step 2: Ligation reaction: 5X DNA Ligase Buffer (3.0 µL); 50% PEG 8000(4.5 µL); 25X Ligation Adaptor (0.6 µL); RNA Ligase (1.5 µL); RNase-free water (0.4 µL). Add 10µL volume of total ligation reaction mix to the tube from step 1, using cycling: Ligation, 16°C for 60 minutes and finished at 4°C. Step 3: Reverse transcription (RT) reaction - following the protocol: 5X RT Buffer (6µL); dNTP Mix (25mM each) (1.2 µL);20X Universal RT Primer (1.5 µL); 10X RT Enzyme Mix (3.0 µL) and RNase-free water (3.3 µL). Add 15ul of the mix RT to the Step 2 tube, with final volume of 30µL, using cycling: Reverse transcription 42°C for 15 minutes; Stop reaction 85°C for 5 minutes and finished at 4°C. Step 4: miR-Amp reaction: 2X miR-Amp Master Mix (25 µL); 20X miR-Amp Primer Mix (2.5 µL); RNase-free water (17.5 µL). After preparing the mix from step 4, add 5µL of the RT reaction product to another 0.2 mL tube, with a final volume of 50µl and incubate the reaction with the following cycling:Enzyme activation 95°C for 5 minutes and a total of 14 cycles (Denature 95°C for 3 seconds and Anneal/Extend 60°C for 30 seconds); Stop reaction 99°C for 10 minutes, finishing the 4°C.

#### 2.5.2 Real-time quantitative PCR

The qRT-PCR reaction was performed as follows: 10µL of TaqMan^®^ Fast Advanced Mix (2X) (Applied Biosystems-Thermofisher- USA PN 4444557); 1 µL TaqMan^®^ Advanced miRNA Assay (20x) (Applied Biosystems-Thermofisher- USA PN A25576); 4µL of RNase-free water and 5 µL of cDNA diluted 1:10. The reaction was performed on the Thermocycler StepOnePlus™ Real-Time PCR System (Thermofisher Scientific, USA) following the cycling conditions: 95 °C for 20s, 40 cycles of 95 °C for 15 s and 60 °C for 20 s, and ending at 4 °C.

### 2.6 Functional enrichment analyses

Validated target genes for differentially expressed miRNAs (DEMs) were identifed using the miRTargetLink 2.0 database (https://ccb-compute.cs.uni-saarland.de/mirtargetlink2/) (19).

Functional enrichment analysis was performed with the Enrichr plataform (https://maayanlab.cloud/Enrichr/) (20, 21) based on the Kyoto Encyclopedia of Genes and Genomes (KEGG) pathways. KEGG terms with adjusted p-value < 0.01 were considered significantly enriched.

#### 2.6.1 MiRNA-target gene network construction

MiRNA-target gene interaction networks were constructed separately for the SITA and NPH groups based on DEMs with KEGG enrichment relevant to T2D. Network visualization was performed using Cytoscape (version 3.10.3) (22).

### 2.7 Statistical analysis

Continuous variables were expressed as mean ± standard deviation (SD), and categorical variables as proportions. Repeated-measures ANOVA followed by Scheffé’s *post hoc* tests was used to assess treatment effects over time. Categorical data were analyzed using chi-square or Fisher’s exact tests, as appropriate. Graphical results are shown as mean ± standard error (SE). For miRNA expression, relative quantification (2^-ΔCt) (23) was used, normalized to cel-miR-39 (18). Depending on data distribution, comparisons of miRNA relative expression were performed using either the non-parametric Mann-Whitney U test or the parametric t-test. A p-value ≤ 0.05 were considered statistically significant.

## 3 Results

This study is an extension of the randomized clinical trial titled “Short and Long-Term Effects of a DPP-4 Inhibitor *Versus* Bedtime NPH Insulin as Add-On Therapy in Patients with Type 2 Diabetes” (11) where 35 adult outpatients with T2D (aged 57 ± 7 years, 57.1% female) inadequately controlled with metformin plus glyburide, were randomized to receive either sitagliptin (100 mg once daily; SITA group) or bedtime NPH insulin (final dose of 11.0 ± 6.7 IU; NPH group). The groups were comparable in terms of glyburide dose (SITA: 17.6 ± 3.1 mg/day; NPH: 18.1 ± 4.1 mg/day; p = 0.68), metformin dose (SITA: 2.4 ± 0.3 g/day; NPH: 2.3 ± 0.6 g/day; p = 0.46), and frequencies of statin (77.8% vs. 64.7%; p = 0.47) or antihypertensive therapy use (77.8% vs. 100%; p = 0.11). These medications remained unchanged throughout the study.

**Figure 1:**
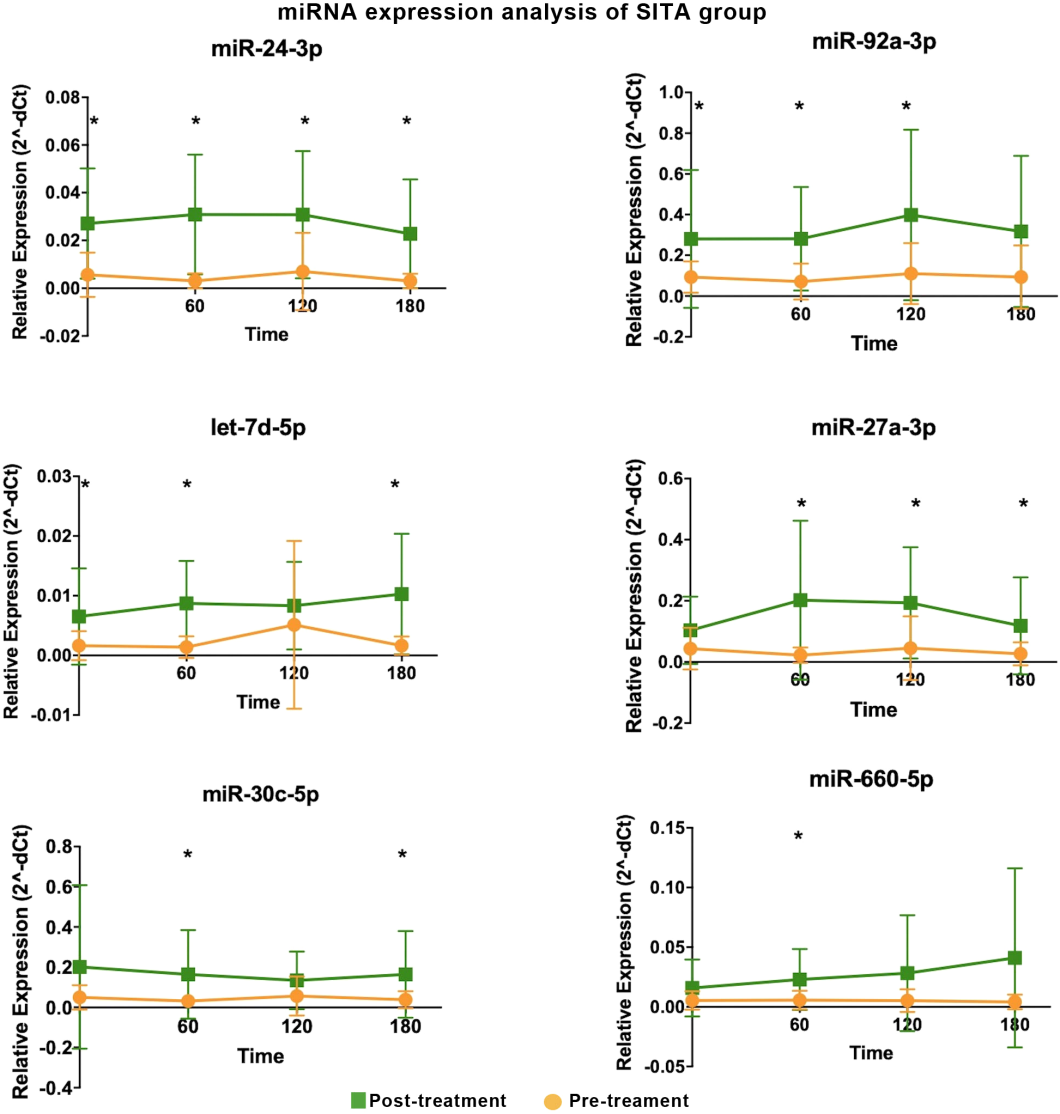
Differentially expressed miRNA in the sitagliptin (SITA) group are shown. Comparative analyses were performed between post- and pre-treatment values at each time point. *Statistical significance was assessed using Mann-Whitney U test, with p < 0.05 considered significant.

**Figure 2:**
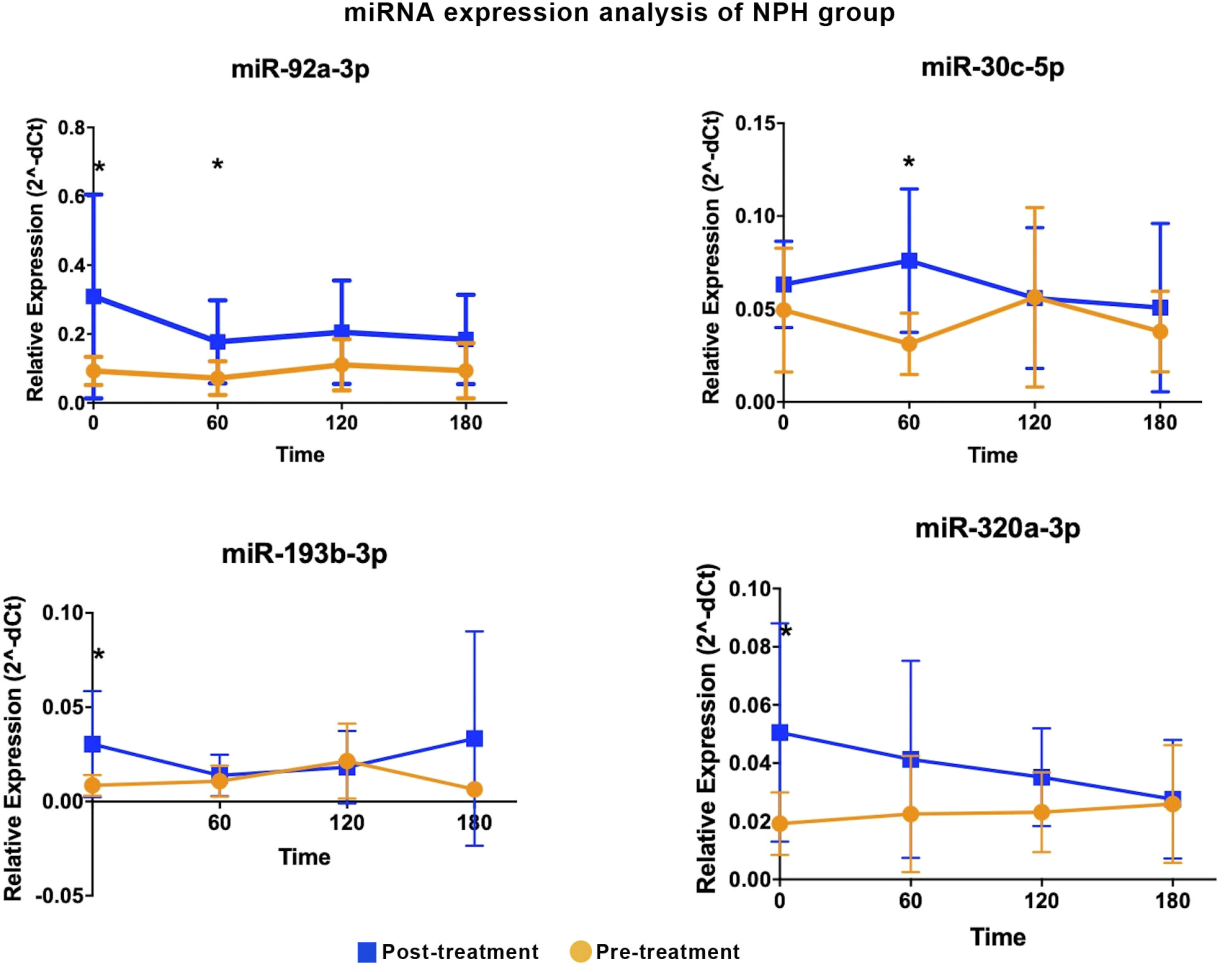
Differentially expressed miRNA in the NPH insulin group are shown. Comparative analyses were performed between post- and pre-treatment values at each time point. * Statistical significance was assessed using Mann-Whitney U test, with p < 0.05 considered significant.

After 6 months of treatment, both sitagliptin and NPH insulin significantly reduced HbA1c values to a similar extent (p<0.001): from 8.1 ± 0.7% to 7.3 ± 0.8% in the SITA group and from 8.1± 0.6% to 7.3 ± 0.7% in the NPH group. Fasting and postprandial (after a standardized 500-kcal mixed meal), active GLP1 concentrations increased 3- to 4-fold at all time points in the SITA group and remained elevated throughout the study. NPH insulin led to reductions in fasting glucose, fasting and postprandial triglyceride, and C-peptide concentrations. Conversely, sitagliptin reduced postprandial insulin and glucagon concentrations. Both treatments suppressed postprandial proinsulin concentrations. NPH insulin was associated with increased post-challenge free fatty acid concentrations and weight gain. A detailed metabolic profile has been reported elsewhere (11) and is presented in **Supplementary Table 2** and **Figures 1** and **2**.

### 3.1 Differential expression of miRNA

Sitagliptin treatment induced upregulation of six miRNAs at various time points (**Figure 1**), with fold-change values ranging from 3 to10 (**Table 1**). Among them, miR-24-3p was consistently upregulated during fasting and across all three postprandial time points. miR-92a-3p, let-7d-5p, miR-27a-3p, miR-30c-5p, and miR-660-5p were differentially expressed at one to three time points. One-hour post-meal, all six miRNAs were significantly upregulated relative to baseline. Notably, miR-24-3p and miR-27a-3p exhibited the highest fold-changes (> 9; **Table 1**).

**Table 1:** Differentially expressed miRNAs in participants treated with sitagliptin (SITA).

Time-interval	miRNA	Relative expression (2^-dCT)		FC (Post-T/Pre-T)	P-value
(minutes)		Mean (Post-T)	Mean (Pre-T)		
Fasting	miR-24-3p	0.03	0.01	4.82	0.00
	miR-let-7d-5p	0.01	0.00	3.94	0.03
	miR-92a-3p	0.28	0.09	3.02	0.04
60	miR-24-3p	0.03	0.00	10.25	0.00
	miR-27a-3p	0.20	0.02	9.05	0.01
	miR-let-7d-5p	0.01	0.00	6.27	0.00
	miR-30c-5p	0.16	0.03	5.23	0.04
	miR-660-5p	0.02	0.01	4.15	0.03
	miR-92a-3p	0.28	0.07	3.93	0.01
120	miR-24-3p	0.03	0.01	4.39	0.01
	miR-27a-3p	0.19	0.05	4.26	0.01
	miR-92a-3p	0.40	0.11	3.60	0.01
180	miR-24-3p	0.02	0.00	7.62	0.00
	miR-let-7d-5p	0.01	0.00	6.25	0.00
	miR-27a-3p	0.12	0.03	4.42	0.03
	miR-30c-5p	0.16	0.04	4.34	0.03

qRT-PCR analyses; FC, fold-change; T, treatment; p-value was obtained using a Mann-Whitney U test.

In the NPH group, four miRNAs were differentially expressed at one- or two-time intervals, with more modest fold-changes (2.43 to 3.57, **Table 2**, **Figure 2**). Specifically, miR-92a-3p, miR-193b-3p, and miR-320a-3p were upregulated during fasting, while miR-30c-5p and miR-92a-3p were upregulated at 1h post-prandial.

**Table 2:** Differentially expressed miRNAs in participants treated with NPH insulin.

Time-interval	miRNA	Relative expression (2^-dCT)		FC (Post-T/Pre-T)	P-value
(minutes)		Mean (Post-T)	Mean (Pre-T)		
Fasting	miR-320a-3p	0.05	0.02	2.64	0.03
	miR-92a-3p	0.31	0.09	3.33	0.03
	miR-193b-3p	0.03	0.01	3.57	0.04
60	miR-30c-5p	0.08	0.03	2.43	0.01
	miR-92a-3p	0.18	0.07	2.48	0.04
120	NS				
180	NS				

qRT-PCR analyses; FC, fold-change; T, treatment; p-value was obtained using a Mann-Whitney U test.

Two miRNAs, miR-92a-3p and miR-30c-5p, showed similar expression patterns in both treatment groups, with increases predominantly observed during fasting and at 60 minutes post-meal.

**Figure 3:**
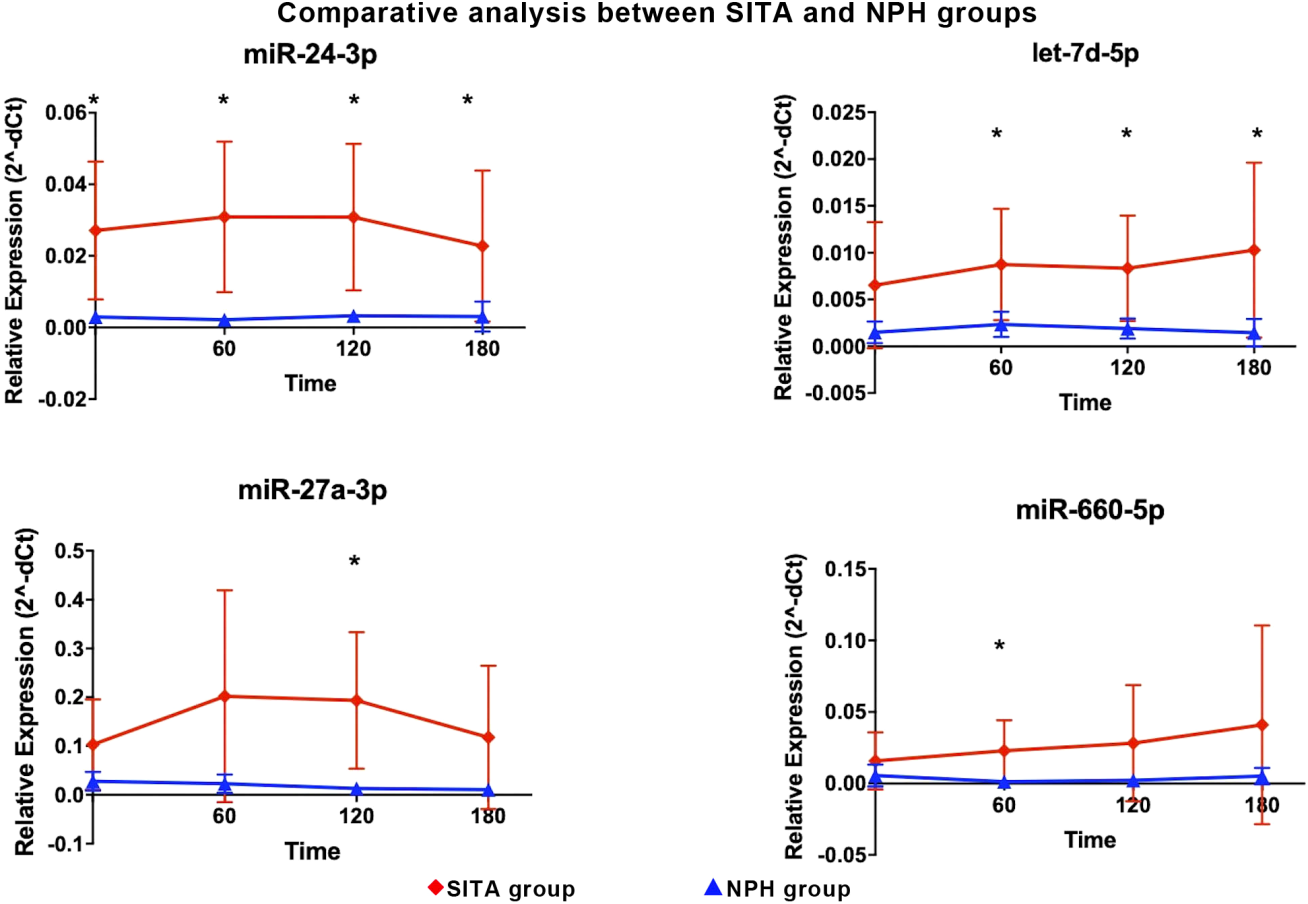
Differentially expressed miRNA between sitagliptin (SITA, red dots) and NPH insulin (NPH, blue dots) groups are shown. Comparative analyses between the groups were performed at each time point. *Statistical significance was assessed using t-test, with p < 0.05 considered significant.

Between-group comparisons revealed that four miRNAs were significantly upregulated in the SITA group (**Figure 3**), with fold-changes ranging from 3.73 to 17.98 (**Table 3**). miR-24-3p was consistently upregulated at all time-points, while let-7d-5p increased between 1 to 3 hours postprandially. Two miRNAs, miR-660-5p and miR-27a-3p were upregulated at 2- and 3-hours, respectively.

**Table 3:** Differentially expressed miRNA between the sitagliptin (SITA) and NPH insulin (NPH) groups.

Time-interval	miRNA	Relative expression (2^-dCT)		FC (SITA/NPH)	P-value
(minutes)		Mean (SITA)	Mean (NPH)		
Fasting	miR-24-3p	0.03	0.00	9.21	0.01
60	miR-660-5p	0.02	0.00	17.98	0.04
	miR-24-3p	0.03	0.00	14.17	0.01
	let7-d-5p	0.01	0.00	3.73	0.04
120	miR-27a-3p	0.19	0.01	14.85	0.01
	miR-24-3p	0.03	0.00	9.36	0.01
	let7-d-5p	0.01	0.00	4.38	0.02
180	miR-24-3p	0.02	0.00	7.41	0.04
	let7-d-5p	0.01	0.00	7.11	0.04

qRT-PCR analyses; FC, fold-change; p-value was obtained using a t-test.

Interestingly, in contrast to the relatively consistent changes in hormonal and metabolic variables, no uniform pattern of miRNA was observed across postprandial time points within each treatment group.

### 3.2 Enrichment analysis of differentially expressed miRNAs

Validated target genes were identified for the differentially expressed miRNAs: 88 for miR-24-3p, 66 for miR-27a-3p, 39 for miR-30c-5p, 35 for miR-92a-3p, 16 for miR-193b-3p, 8 for let-7d-3p, 1 for miR-660-5p (**Supplementary Table 4**) and none for miR-320a-3p.

Two gene sets were constructed for functional analysis, comprising validated targets of the six and four differentially expressed miRNAs in the SITA and NPH groups, respectively. This yielded 222 genes for the SITA group and 88 for the NPH group.

**Figure 4:**
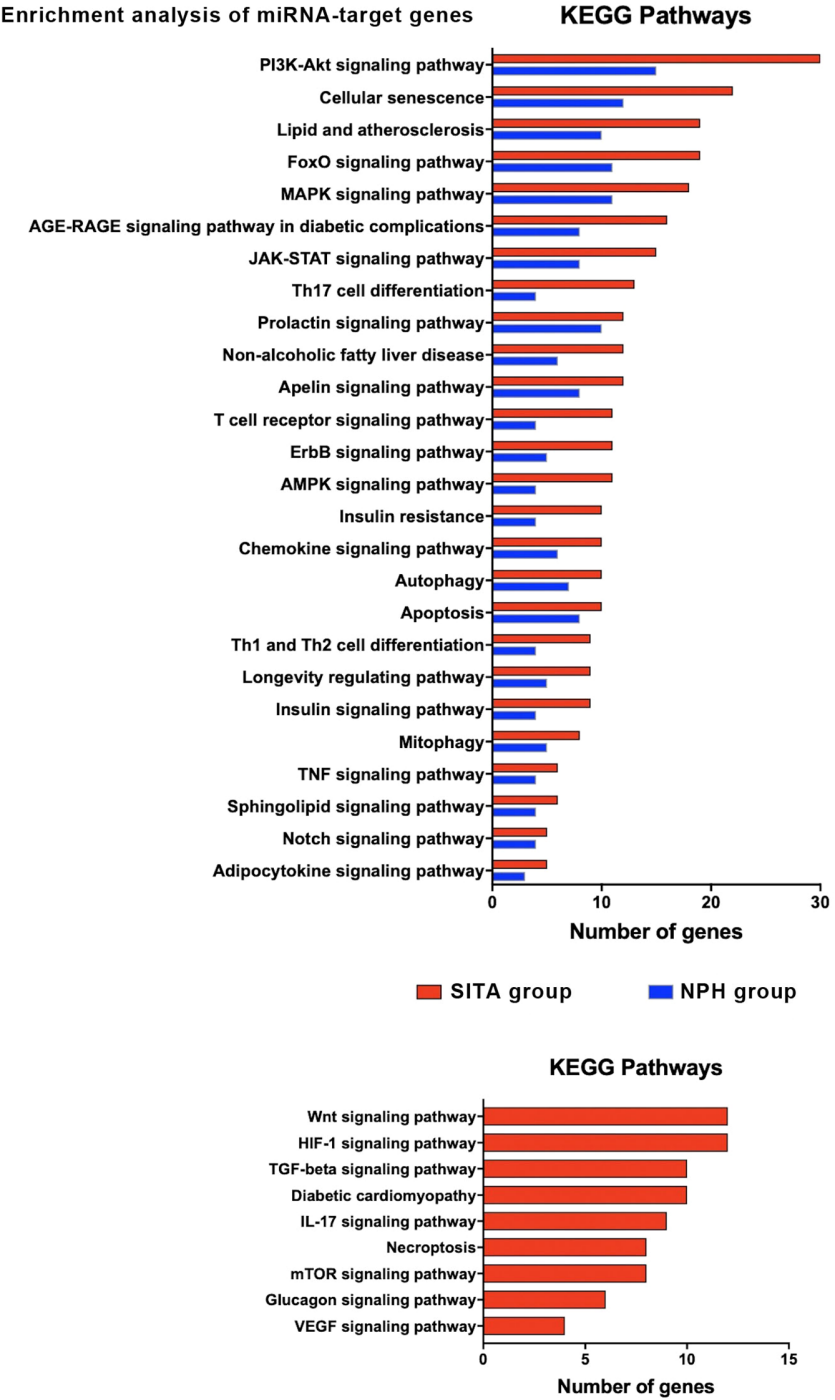
Functional enrichment analysis of target genes for differentially expressed miRNAs (DEM) in the sitagliptin (SITA) and NPH insulin (NPH) groups. KEGG pathway analysis for target genes of the six DEMs (miR-24-3p, miR-27a-3p, miR-30c-5p, miR92a-3p, miR-660-5p, and let-7-5p) identified in the SITA group (red bars), and three DEMs (miR-92a-3p, miR-30c-5p, and miR-193b-3p) identified in the NPH group (blue bars). Only KEGG terms with adjusted p < 0.05 were considered significant. The histograms depict pathways related to T2D.

KEGG pathway enrichment analysis revealed 26 shared pathways and nine unique to the SITA group (**Figure 4**, **Supplementary Table 2**). The number of genes mapped to each pathway varied between groups.

In both groups, enriched pathways were primarily related to signal transduction, cellular senescence, lipid metabolism and atherosclerosis, Th17 cell differentiation, insulin resistance, autophagy, and apoptosis. However, seven signaling pathways - Wnt, HIF1, TGF beta, IL17, mTOR, glucagon, and VEGF - along with pathways involved in diabetic cardiomyopathy and necroptosis, were exclusively enriched in the SITA group (**Figure 4**).

**Figure 5:**
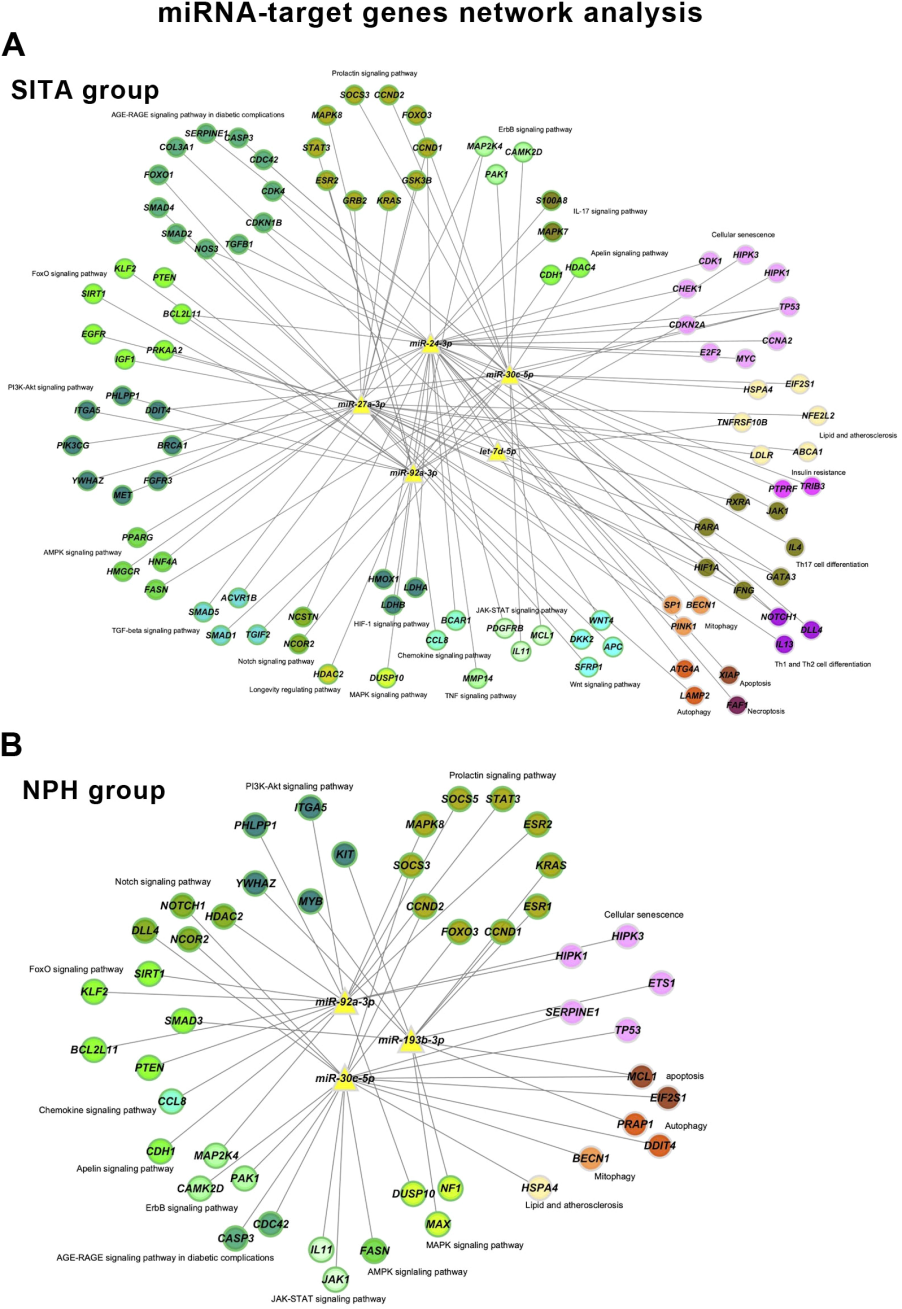
miRNA-target gene networks. Networks are shown for the sitagliptin (SITA) **(A)** and NPH insulin (NPH) **(B)** groups. Nodes are color-coded according to enriched KEGG pathways and green borders indicate genes involved in signaling pathways.

Network analyses summarized the interactions among miRNAs, their target genes, and biological pathways based on the highest odds ratios from the enrichment analysis (**Figure 5**, **Supplementary Table 5**).

The sitagliptin network included five miRNAs, 102 genes, and 237 interactions, whereas the insulin network comprised three miRNAs, 48 genes, and 90 interactions. Sixteen KEGG terms were present in both networks, but the SITA group showed broader involvement in signaling pathways, cellular senescence, lipid and atherosclerosis, mitophagy, and autophagy.

## 4 Discussion

To our knowledge, this is the first study to compare the effects of sitagliptin and bedtime NPH insulin on circulating miRNAs expression in individuals with long-standing T2D. Our findings demonstrate that both therapies modulate serum miRNA levels, with sitagliptin exerting a broader and more sustained influence across multiple postprandial time points. In contrast, bedtime NPH insulin was associated with more limited changes, primarily restricted to fasting and early postprandial states.

Among the 10 miRNAs evaluated, seven were upregulated following treatment, with sitagliptin accounting for the majority of these changes. Two miRNAs - miR-92a-3p and miR-30c-5p – were similarly upregulated in both treatment arms, suggesting the involvement of shared pathways, likely related to the observed metabolic improvements. miR-30c-5p has been implicated in reducing hyperlipemia and atherosclerosis in a murine model (24), inhibiting pyroptosis (25) and apoptosis in neural progenitor cells, (26) and exerting renoprotective effects by attenuating high glucose-induced oxidative stress, inflammation, and extracellular matrix accumulation (27).

As for miR-92a-3p, its target genes include members of the SMAD family, key mediators of the transforming growth factor beta 1 (TGFB1) signaling pathway, thus, its upregulation may be associated with reduced fibrosis (28) and inflammation (29). Interestingly, miR-92a-3p expression has been reported to decrease following interventions known to improve insulin sensitivity, such as metformin (6), pioglitazone (7) and gastric by-pass surgery (30). This contrasts with the upregulation observed in the present study, which may reflect the fact that bedtime NPH insulin and sitagliptin improve glycemic control primarily through non-insulin-sensitizing mechanisms. One possibility is the report of Xu et al. (31) which showed that mir-235/miR-92, by suppressing Wnt signaling, upregulates several autophagy genes, enhances autophagy, and promotes longevity, thereby supporting glucose metabolism. Alternatively, the observed increase in miR-92a-3p may reflect broader metabolic changes rather than being directly related to the specific mechanisms of action of the drugs.

Four miRNAs - miR-24-3p, miR-27a-3p, let-7d-5p, and miR-660-5p - were exclusively upregulated following sitagliptin treatment. Among these, miR-24-3p and miR-27a-3p have been associated with anti-inflammatory effects (32, 33). In addition, miR-27a-3p has been shown to enhance muscle glycogen storage and alleviate insulin resistance by modulating FOXO1 signaling and downregulating gluconeogenic enzymes (34). Let-7d-5p has demonstrated the ability to attenuate neuroinflammation (35) and the progression of atherosclerosis, likely through inhibition of NF-kB-mediated inflammation and vascular smooth muscle cells proliferation (36), suggesting a potential protective role against vascular complications. Interestingly, circulating let-7d-5p was downregulated in individuals with hepatic steatosis whereas hepatic expression was increased and correlated with insulin resistance (37), underscoring the complex and tissue-specific regulation of miRNAs.

Circulating miR-660-5p has previously been reported to be downregulated in obese children with insulin resistance and to correlate with changes in glycemia and insulinemia (Δ0–120 min) during an oral glucose tolerance test in obese children without insulin resistance (38). The regulation of this miRNA likely depends on distinct metabolic variables, as suggested by its divergent responses under various insulin-related conditions: it was downregulated during hyperinsulinemic-euglycemic clamp, upregulated in response to insulin plus intralipid infusion - a model known to exacerbate insulin resistance (16) - and remained unchanged following bedtime NPH insulin therapy in the present study, while it was upregulated exclusively after sitagliptin treatment.

Two miRNAs - miR-320a-3p and miR-193b-3p - were exclusively upregulated in the fasting state following bedtime NPH insulin treatment. The miR-320a has been reported to inhibit glucagon secretion (39, 40), and to be negatively correlated with fasting plasma glucose and insulin resistance in obese individuals with metabolic syndrome (41). In contrast, it was positively associated with insulin resistance in healthy individuals without T2D (42) and with fasting glucose in individuals with T2D (39). Furthermore, miR-320a-3p was downregulated in participants treated with metformin in the Diabetes Prevention Program (DPP) (6) and in individuals with T2D undergoing gastric bypass surgery (43). Experimental data also indicate a role for miR-320a in beta-cell dysfunction in high-fat diet-fed mice (44). These findings suggest that its downregulation may be beneficial in the context of T2D. Therefore, its upregulation in the fasting state of individuals receiving bedtime NPH insulin in the present study is intriguing and may reflect a context-dependent response whose physiological significance warrants further investigation.

Circulating miR-193b-3p has been reported to be upregulated in newly diagnosed, untreated individuals with T2D, and is believed to directly influence glucose metabolism by upregulating the transcription factor FOXO1, thereby enhancing the expression of gluconeogenic enzymes and promoting hepatic glucose production (12). Elevated levels of this miRNA have also been observed in individuals with prediabetes and in glucose-intolerant mice, with a reduction following chronic exercise intervention (45). Given that a key mechanism by which bedtime insulin suppresses hepatic glucose production involves the inactivation of FOXO1, leading to reduced gluconeogenesis (46) the upregulation of miR-193b-3p in this context is unexpected. The fact that all study participants were taking metformin, which is known to attenuate FOXO1 activity (47), makes the interpretation of this finding - observed only in the bedtime NPH insulin-treated group - even more challenging. Nonetheless, it may represent a compensatory mechanism to preserve glucose homeostasis under specific metabolic conditions.

Discrepancies with previous studies may be attributed to several factors, including differences in sample collection timing (e.g., fasting vs. postprandial), the biological source of miRNAs (e.g., serum vs. extracellular vesicles, diabetes duration, degree of glycemic control and the statistical approaches used for data analysis. Additionally, the fact that our participants were already being treated with metformin and glyburide may have influenced circulating miRNA levels. These discrepancies highlight the complexity of studying miRNAs, as their expression appears to be modulated by a wide range of metabolic and contextual variables. This inherent variability poses a challenge for establishing miRNAs as reliable biomarkers in a multifactorial condition such as T2D.

This study has several limitations that should be acknowledged. The relatively small sample size may limit the generalizability of the findings, and the lack of a drug-naïve control group prevents us from fully isolating the effects of the interventions from those of prior treatments. Moreover, the observational nature of the miRNA analyses does not allow for causal inferences.

Despite these limitations, the predicted gene targets of miRNAs modulated by both sitagliptin and NPH insulin treatment converged on key signaling pathways associated with improved glucose control. Notably, pathways such as PI3K-Akt, MAPK, insulin, prolactin, ErB, apelin and lipid and atherosclerosis signaling are involved in glucose metabolism, protein synthesis, and energy metabolism. The activation of the Foxo signaling pathway observed in our analysis may have been influenced by metformin use among study participants.

In addition to metabolic pathways, immunomodulatory and inflammatory signaling emerged as relevant targets. These included the prolactin pathway, Th17 differentiation, T cell receptor, chemokines, Th1-Th2 cell, TNF, and JAK-STAT pathways. These findings may reflect either postprandial inflammatory responses or underlying diabetes-related complications, particularly given the long disease duration in our cohort. Activation of AGE-RAGE and cellular senescence pathways was also observed, consistent with chronic metabolic stress.

Pathways related to cell cycle regulation, such as autophagy, apoptosis, and mitophagy, may also be secondary to the metabolic derangements or complications of longstanding diabetes. Interestingly, glucagon signaling was exclusively enriched in the SITA group, suggesting a potentially distinct mechanism of action or feedback regulation related to incretin-based therapy.

It is noteworthy that many of the genes targeted by the identified miRNAs (**Supplementary Table 4**) have been predominantly studied in contexts such as cancer, apoptosis, cellular development and differentiation, inflammation, cellular stress, and signaling pathways involved in insulin resistance. These regulatory pathways often intersect with mechanisms of metabolic homeostasis and the pathophysiology of diabetes.

In our initial analysis, we focused on genes targeted by two or more miRNAs, including *BCL2L11, CCND1, DNMT1, FBXW7, GATA3, IFNG, IFNGR, KRAS, MAP2K4, MAPK14, MCL1, NOTCH1, SP1*, and *TP53*. Among these, several are involved in key cellular processes: *BCL2L11* and *TP53* are associated with apoptosis; *MAP2K4*, *MAPK14*, and *SP1* are linked to inflammatory signaling and cellular stress, while *IFNG* and its receptor (*IFNGR****)*** participate in inflammatory cytokine signaling implicated in beta-cell apoptosis and insulin resistance.

Specifically, regarding genes related to insulin resistance pathway (**Supplementary Table 3**), we highlight *SOCS3, GSK3B, STAT3, PTEN, TRIB3, FOXO1*, and *PTPRF*, along with *MAPK8*, which plays a role in metabolic stress and inflammation. Many of these genes also participate in broader regulatory networks, including *FASN* and *GRB2* in insulin signaling pathways, as well as *JAK1, ITGA5, FOXO3* in the PI3K-Akt signaling pathway.

Collectively, these findings suggest that the modulation of gene expression by miRNAs may contribute to improved metabolic control and represent a potential therapeutic avenue in the treatment of T2D, particularly through targeting key genes involved in insulin resistance and inflammation.

## 5 Conclusion

Adjunctive therapy with sitagliptin and bedtime NPH insulin was associated with metabolic improvement and distinct modulation of circulating miRNAs, with sitagliptin influencing a broader spectrum of miRNA expression. The upregulated miRNAs are involved in pathways related to insulin signaling, inflammation, and cellular homeostasis. These findings support the hypothesis that sitagliptin exerts pleiotropic effects beyond glycemic control.

## Data Availability

The original contributions presented in the study are included in the article/**Supplementary Material**. Further inquiries can be directed to the corresponding author/s.
