# Supplementary material for: MicroRNAs modulated by DPP-4 inhibitor and bedtime NPH insulin therapy in individuals with type 2 diabetes
Source: Front Endocrinol (Lausanne). 2025 Nov 7;16:1706951. doi: 10.3389/fendo.2025.1706951 (PMC12634347; doi:10.3389/fendo.2025.1706951)
Supplement: Supplementary file 2 [file Table2.docx]

| Suppl 2- Clinical and biochemical characteristics of 35 patients with type 2 diabetes at baseline and after 6 and 12 months | | | | | | |
| --- | --- | --- | --- | --- | --- | --- |
| of sitagliptin or bedtime NPH insulin therapy | |  |  |  |  |  |
| Variable |  | **SITAGLIPTIN** | |  | **NPH INSULIN** |  |
|  | Baseline | After 6 months | After 12 months | Baseline | After 6 months | After 12 months |
| Number of patients | 18 | 18 | 15 | 17 | 17 | 16 |
| Female / Male | 09/set |  |  | 11:06 |  |  |
| Age (years) | 55.1 ± 6.7 |  |  | 58.4 ± 6.9 |  |  |
| Diabetes duration (years) | 10.9 ± 5.8 |  |  | 10.9 ± 7.5 |  |  |
| Weight (kg) | 69.3 ± 12.0 | 69.9 ± 11.8 | 68.2 ± 12.0 | 73.4 ± 9.7 | 74.3 ± 10.5a | 74.4 ± 10.9a |
| BMI (kg/m²) | 26.5 ± 2.7 | 26.8 ± 2.6 | 26.6 ± 3.1 | 27.5 ± 2.5 | 27.8 ± 2.8 | 27.9 ± 2.8 |
| HbA1c (%) | 8.0 ± 0.6 | 7.3 ± 0.8a | 7.3 ± 0.8a | 8.1 ± 0.7 | 7.3 ± 0.7a | 7.3 ± 0.9a |
| HbA1c (mmol/mol) | 64.5 ± 17.3 | 56.6 ± 14.8a | 56.1 ± 14.7a | 65.3 ± 16.5 | 56.7 ± 16.1a | 56.8 ± 13.1a |
| Glucose (mg/dL) | 136.5 ± 30.0 | 125.6 ± 33.4 | 115.7 ± 51.5 | 159.2 ± 38.0 | 111.6 ± 30.4a | 127.5 ± 48.3a |
| Triglycerides (mg/dL) | 139.1 ± 63.5 | 144.1 ± 66.2 | 154.0 ± 85.1 | 152.6 ± 80.5 | 130.1 ± 85.3 | 110.7 ± 52.3a |
| Total cholesterol (mg/dL) | 171.7 ± 35.1 | 174.7 ± 33.3 | 164.2 ± 36.9 | 186.8 ± 29.0 | 185.2 ± 42.0 | 181.6 ± 34.9 |
| LDL-cholesterol (mg/dL) | 96.6 ± 32.0 | 101.9 ± 26.7 | 85.5 ± 33.0 | 114.1 ± 21.0 | 121.8 ± 25.6 | 112 ± 29.0 |
| HDL-cholesterol (mg/dL) | 46.2 ± 9.9 | 43.4 ± 8.3 | 41.5 ± 17.2 | 42.0 ± 10.2 | 45.5 ± 11.8 | 43.0 ± 11.0 |
| Active GLP-1 (pmol/L) | 5.0 ± 2.3 | 15.3 ± 10.3a | 16.6 ± 15.9a | 5.0 ± 2.4 | 5.6 ± 2.1 | 6.3 ± 3.6 |
| NEFA (mEq/L) | 0.61 ± 0.25 | 0.64 ± 0.18 | 0.60 ± 0.29 | 0.51 ± 0.21 | 0.45 ± 0.17 | 0.46 ± 0.12 |
| Insulin (µU/mL) | 17.6 ± 5.3 | 21.9 ± 8.9 | 22.2 ± 9.8 | 21.5 ± 11.7 | 31.7 ± 19.4 | 36.3 ± 24.4a |
| C peptide (ng/mL) | 2.7 ± 0.9 | 2.9 ± 1.0 | 3.0 ± 1.3 | 3.4 ± 1.3 | 2.9 ± 1.3 | 2.6 ± 1.2a |
| Proinsulin (pmol/L) | 36.7 ± 28.1 | 34.6 ± 32.1 | 49.8 ± 51.6 | 39.0 ± 29.3 | 40.2 ± 29.2 | 49.2 ± 48.1 |
| Glucagon (pg/mL) | 84.0 ± 34.2 | 86.5 ± 36.6 | 85.5 ± 24.5 | 83.8 ± 33.8 | 87.8 ± 38.4 | 84.9 ± 26.0 |
| Proinsulin/C peptide ratio | 13.5 ± 8.3 | 11.9 ± 10.4 | 16.9 ± 16.0a,b | 11.0 ± 5.7 | 13.3 ± 6.9a | 16.4 ± 10.8a,b |
| Data are expressed as mean±SD. BMI, body mass index. HbA1c, glycated hemoglobin. GLP-1, glucagon-like peptide 1. | | | | | | |
| NEFA, free fat acids. |  |  |  |  |  |  |
| a p<0.05 for the difference between before and after treatments. | | | |  |  |  |
| b p<0.05 for the difference between 6 and 12 months of each treatment | | | |  |  |  |
|  |  |  |  |  |  |  |

| Sitagliptin | NPH Insulin |
| --- | --- |
|  |  |
|  |  |
|  |  |
|  |  |
|  |  |
|  |  |

**Fig 1**- Changes in blood glucose, glucagon-like peptide -1(GLP-1), insulin, C-peptide, proinsulin levels and proinsulin/C peptide ratio during the meal test before (•) and after six (ο) and twelve ( ) months of sitagliptin and NPH insulin therapies; P<0.05 for baseline vs 6 months(**α**); baseline vs 12 months (**β**); 6 months vs 12 months (**δ**) of each therapy

| Sitagliptin | NPH Insulin |
| --- | --- |
|  |  |
|  |  |
|  |  |

**Fig 2**- Changes in blood glucagon, free fat acids (NEFA), and triglyceride levels during the meal test before (•) and after six (ο) and twelve (
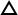
) months of sitagliptin and NPH insulin therapies; P<0.05 for baseline vs 6 months(**α**); baseline vs 12 months (**β**); 6 months vs 12 months (**δ**) of each therapy
