# Supplementary material for: MicroRNAs modulated by DPP-4 inhibitor and bedtime NPH insulin therapy in individuals with type 2 diabetes
Source: Front Endocrinol (Lausanne). 2025 Nov 7;16:1706951. doi: 10.3389/fendo.2025.1706951 (PMC12634347; doi:10.3389/fendo.2025.1706951)
Supplement: Supplementary file 3 [file Table3.docx]

**Supplementary Table 3**- Description of the evaluated miRNAs- TaqMan^™^ Advanced miRNA Assay (PN A25576- Thermofisher Scientific)

| **mature miRNA** | **Assay ID** | **Mature miRNA Sequence** | **miRBase Accession Number(s)** |
| --- | --- | --- | --- |
| **hsa-miR-24-3p** | 477992_mir | UGGCUCAGUUCAGCAGGAACAG | MIMAT0000080 |
| **hsa-miR-27a-3p** | 478384_mir | UUCACAGUGGCUAAGUUCCGC | MIMAT0000084 |
| **hsa-miR-30c-5p** | 478008_mir | UGUAAACAUCCUACACUCUCAGC | MIMAT0000244 |
| **hsa-miR-92a-3p** | 477827_mir | UAUUGCACUUGUCCCGGCCUGU | MIMAT0000092 |
| **hsa-miR-193b-3p** | 478314_mir | AACUGGCCCUCAAAGUCCCGCU | MIMAT0002819 |
| **hsa-miR-199a-3p** | 477961_mir | ACAGUAGUCUGCACAUUGGUUA | MIMAT0000232 |
| **hsa-miR-320a-3p** | 478594_mir | AAAAGCUGGGUUGAGAGGGCGA | MIMAT0000510 |
| **hsa-miR-335-5p** | 478324_mir | UCAAGAGCAAUAACGAAAAAUGU | MIMAT0000765 |
| **hsa-miR-532-5p** | 478151_mir | CAUGCCUUGAGUGUAGGACCGU | MIMAT0002888 |
| **hsa-miR-660-5p** | 478192_mir | UACCCAUUGCAUAUCGGAGUUG | MIMAT0003338 |
| **hsa-let-7d-5p** | 478439_mir | AGAGGUAGUAGGUUGCAUAGUU | MIMAT0000065 |
|  |  |  |  |
| **Spike-in** |  |  |  |
| **cel-miR-39-3p** | [478293_mir](https://www.thermofisher.com/order/genome-database/details/microrna/478293_mir) | UCACCGGGUGUAAAUCAGCUUG | MIMAT0000010 |
|  |  |  |  |
|  |  |  |  |
